# The African disability scooter: efficiency testing in paediatric amputees in Malawi

**DOI:** 10.3109/17483107.2014.932851

**Published:** 2014-10-15

**Authors:** Verona Beckles, Jennifer L. McCahill, Julie Stebbins, Nyengo Mkandawire, John C. T. Church, Chris Lavy

**Affiliations:** ^a^ Beit Cure International Hospital, Blantyre, Malawi; ^b^ Oxford Gait Laboratory, Nuffield Orthopaedic Centre, Headington, Oxford, UK; ^c^ Department of Surgery, College of Medicine, University of Malawi, Blantyre, Malawi; ^d^ Emeritus Consultant Surgeon, Buckinghamshire, England; ^e^ Nuffield Department of Rheumatology and Orthopaedic Surgery, Nuffield Orthopaedic Centre, Headington, Oxford, UK

**Keywords:** Amputee, mobility, oxygen cost, scooter

## Abstract

*Purpose*: The African Disability Scooter (ADS) was developed for lower limb amputees, to improve mobility and provide access to different terrains. The aim of this study was to test the efficiency of the ADS in Africa over different terrains. *Method*: Eight subjects with a mean age of 12 years participated. Energy expenditure and speed were calculated over different terrains using the ADS, a prosthetic limb, and crutches. Repeated testing was completed on different days to assess learning effect. *Results*: Speed was significantly faster with the ADS on a level surface compared to crutch walking. This difference was maintained when using the scooter on rough terrain. Oxygen cost was halved with the scooter on level ground compared to crutch walking. There were no significant differences in oxygen consumption or heart rate. There were significant differences in oxygen cost and speed between days using the scooter over level ground, suggesting the presence of a learning effect. *Conclusions*: This study demonstrates that the ADS is faster and more energy efficient than crutch walking in young individuals with amputations, and should be considered as an alternative to a prosthesis where this is not available. The presence of a learning effect suggests supervision and training is required when the scooter is first issued.

**Implications for Rehabilitation**
- The African Disability Scooter:
- is faster than crutch walking in amputees;
- is more energy efficient than walking with crutches;
- supervised use is needed when learning to use the device;
- is a good alternative/adjunct for mobility.

## Introduction

Amputations are common in many resource-poor countries. They tend to occur as a consequence of injury, infection, tumours and neglected congenital conditions [1]. The exact number of amputations each year in developing countries is not known. Banza et al. [1] reported 1500 children's orthopaedic and plastic surgery operations performed each year, with an average of nine amputations per year in the five-year period studied. The study was conducted at a charitable, elective admissions hospital in Blantyre, Malawi. In the West, it is estimated that approximately four new paediatric amputees are referred to limb fitting centres each year; this is less than half of the amputations performed in developing countries [2]. Lower limb amputees face many challenges in order to mobilise independently. Availability of prostheses is often problematic in developing countries, posing real challenges for children accessing school, participating in activities of daily living and contributing to domestic responsibilities.

**Figure 1.**
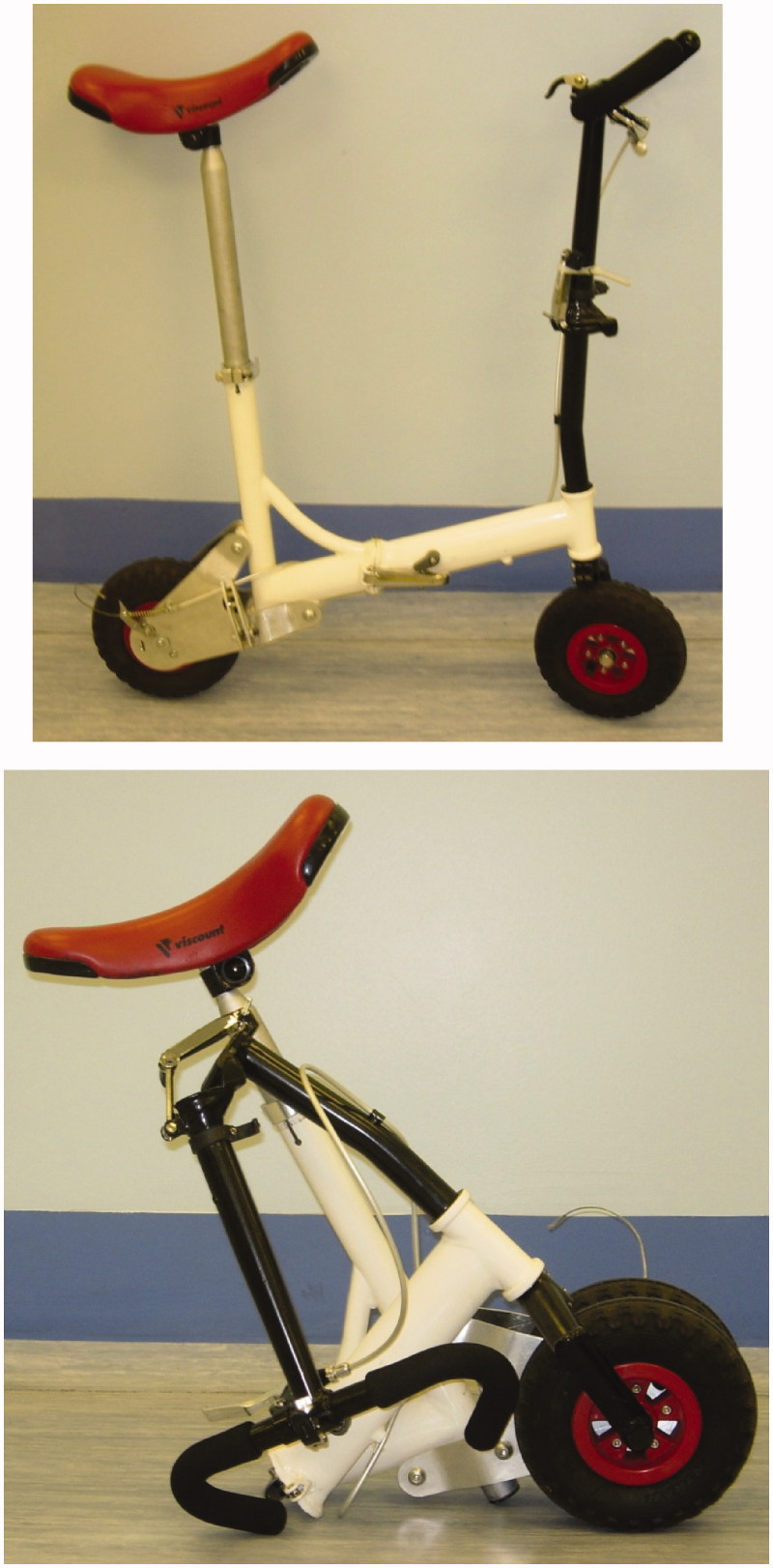
The African Disability Scooter.

The African Disability Scooter (ADS) was developed to improve mobility for amputees, with these challenges in mind (Figure 1). As stated in a previous study, the ADS was designed to be “robust on uneven terrain, light weight, manoeuvrable, portable, manufactured at low cost with materials local to the region, and able to be mass-produced” [3]. Preliminary testing of the ADS was conducted using healthy adults with a “simulated” amputation. This study indicated that the scooter was twice as efficient and twice as fast than using crutches over level ground [3].

However, the ADS is yet to be assessed in the environment for which it was designed. In addition, it is unknown if the ADS improves mobility for subjects with amputations. Therefore, the aim of this study was to assess the speed and energy efficiency of mobility when using the ADS, compared to walking with crutches and walking with a prosthesis, on different terrains, in children and adolescents who have had a unilateral lower limb amputation.

It was hypothesized that the ADS would improve speed and energy efficiency compared to walking with crutches, and provide comparable speed and efficiency to walking with a customised prosthetic limb.

## Participants and methods

Ethical approval for the study was obtained through the College of Medicine Research and Ethics Committee (COMREC), College of Medicine Malawi, University of Malawi.

The study was conducted at a charitable paediatric orthopaedic teaching hospital in Blantyre, Malawi, during school holidays. The subjects were recruited from the hospital records and a local rehabilitation centre with prosthetic records. Eight subjects (six female and two male) with a mean age of 12 years were recruited. All subjects had a unilateral lower limb amputation and were required to be at least 130 cm tall to account for the lowest possible seat height on the scooter. Two subjects had above-knee amputations and six had below knee amputations. The cause of amputation was either congenital or traumatic. The time since amputation was between 2 and 10 years, and all participants were regular prosthesis users. Informed consent was obtained from their guardians. Communication with the participants and their guardians was performed using translators who translated from Chichewa to English and English to Chichewa as necessary.

The study took place over a 14-day period with 6 days of data collection. After a preliminary training and testing session, the participants were allowed daily supervised access to the scooter to develop their confidence with it as a mobility aid. Mini races and play activities were also arranged (Table 1).

**Table 1.** Data collection schedule.

Day	Mobility method around course
1	**Testing**: Scooter on level surface with prosthetic leg
2	**Testing**: Walking on level surface with prosthetic leg **Testing**: Scooter on level surface with prosthetic leg
3	**Testing**: Walking on level surface with axilla crutches
4	Scooter on level surface with prosthetic leg
5	Activities/games on scooter
6	Activities/games on scooter
7	REST DAY
8	Activities/games on scooter
9	Activities/games on scooter
10	Activities/games on scooter
11	Scooter on level surface without prosthetic leg
12	**Testing**: Scooter on level surface with prosthetic leg **Testing**: Scooter on level surface without prosthetic leg
13	**Testing**: Scooter on uneven surface with prosthetic leg
14	**Testing**: Scooter on uneven surface without prosthetic leg

Bold indicates test conditions.

A figure of eight track, 65 m in length, was measured out on a level tarmac surface for testing on days 1, 2, 3 and 12, and on an uneven grass surface on days 13 and 14. The subjects were given the ADS and instructed to adjust the seat height until they felt “comfortable”. All subjects were measured for appropriate crutch height using Mulley's guidelines ± 2.5 cm [4] and received instructions on how to use the crutches. The K4b^2^ system (Cosmed, Rome, Italy) was fitted to the subject according to manufacturer's instructions [5]. This allowed calculation of energy expenditure by measuring the concentration and volume of expired oxygen and carbon dioxide.

Subjects were tested using the ADS (on both level and uneven ground), axilla crutches (level ground) and their prosthetic leg (level ground) as per Table 1. Real time data of heart rate and oxygen consumption was viewed and recorded on a laptop. Prior to each data collection, the subjects were instructed to remain quietly seated until resting heart rate was obtained.

The participants were then instructed to complete a minimum of three laps around the fixed course, ensuring that a steady-state heart rate was achieved. Following this, the participant would return and sit quietly until their heart rate returned to a resting state.

It should be noted that for the scooter, the same measurements were taken on day 1, 2 and 12 (with the subject's prosthetic leg in place) in order to assess the presence of any learning effect.

Following each trial of the ADS, the participants used a visual analogue scale to report their perceived comfort, ease of use and stability of the scooter.

### Measurements and derived calculations

Speed was assessed by measuring the time taken to complete each lap of the course using a stop watch. Energy expenditure and heart rate were calculated using the K4b^2^ system. Oxygen consumption (VO_2_) was defined by the following equation:

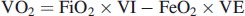

where

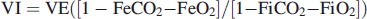

FiO_2_=%O_2_ in inspired air.
FiCO_2_=%CO_2_ in inspired air.
VI=inhaled volume per minute.
VE=expired volume per minute
FeO_2_=%O_2_ in expired air.
CO_2_=%CO_2_ in expired air.
Energy cost was calculated by dividing VO_2_ by speed.

Statistics were calculated using SPSS software (SPSS Inc, version 13). Single-factor analysis of variance (ANOVA) was used to calculate differences between conditions (ADS level ground, ADS uneven ground, walking with crutches and walking with prosthetic limb) with a *posthoc* Tukey test (*p* < 0.05). The scooter testing from the final testing sessions (days 12 to 14, without prosthetic limb in place) was used for testing between conditions.

## Results

**Figure 2.**
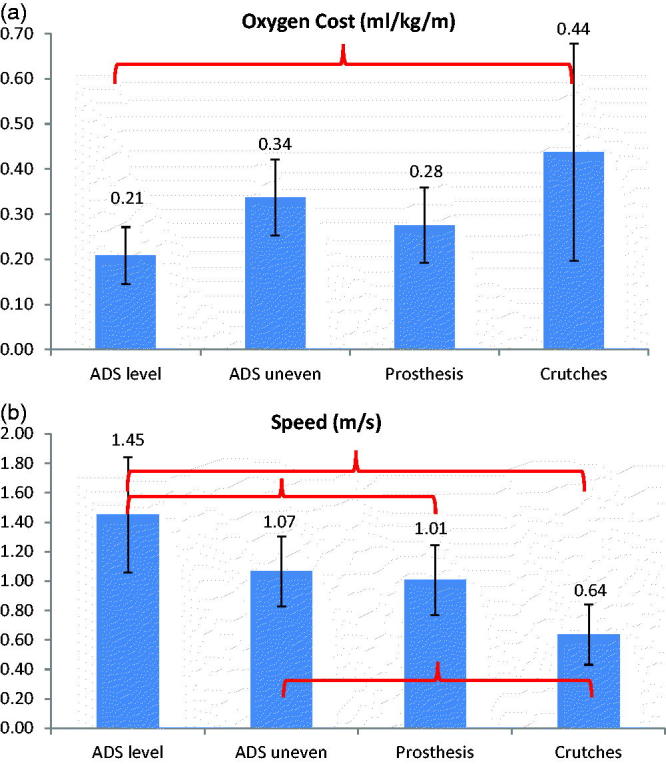
(a) Difference in oxygen cost between conditions. Brackets indicate significant differences. (b) Difference in speed between conditions. Brackets indicate significant differences.

One subject was unable to complete the testing due to other commitments, so the data from the remaining seven subjects is reported here. There was a significant difference in oxygen cost between the ADS on level ground and crutches (*p* = 0.02). Oxygen cost was halved when using the scooter compared to walking with crutches (Figure 2a). There were no other significant differences in oxygen cost between conditions.

Speed was significantly faster with the scooter compared to crutches on level ground (*p* < 0.01) (Figure 2b) and on uneven ground (*p* = 0.04). Over level ground, the scooter was also significantly faster than walking on a customised prosthesis (*p* = 0.03).

**Figure 3.**
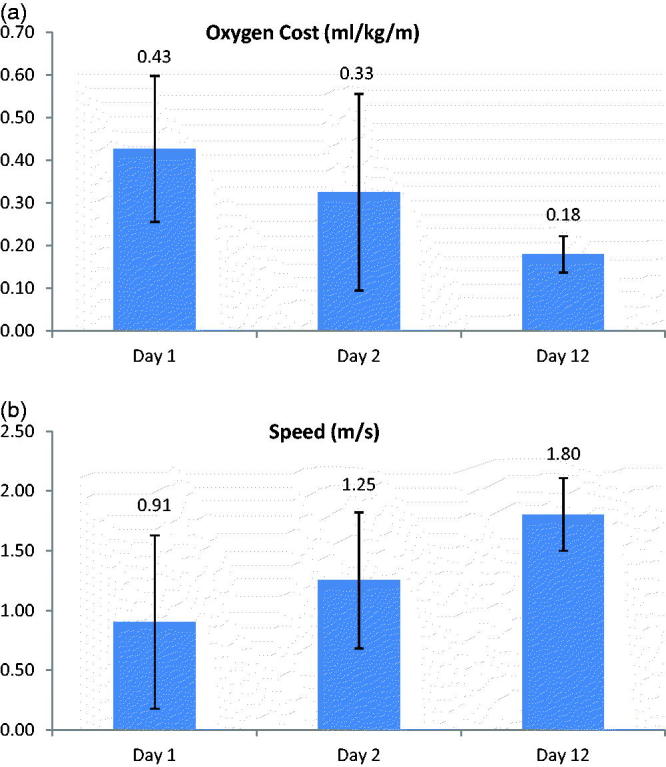
(a) Differences in oxygen cost between repeated testing on different days. Testing was completed with the subject's prosthetic limb in place. (b) Differences in speed between repeated testing on different days. Testing was completed with the subject's prosthetic limb in place.

There was a significant difference in oxygen cost between days over level ground (*p* = 0.04) (Figure 3a). There was also a significant difference (*p* = 0.02) showing increasing speed following repeated use (Figure 3b). This indicates the presence of a learning effect when using the scooter. There were no significant differences in oxygen consumption (VO_2_) or heart rate between days.

In terms of the comfort, safety and stability reported by the participants using a visual analogue scale, initially some participants scored the ADS low especially for safety and stability. However, with repeated use these scores increased. All participants found the ADS comfortable (VAS > 7) throughout the testing period, and 5/7 felt safe and stable on it throughout the testing period (VAS > 7).

## Discussion

This study assessed the effectiveness of a scooter specifically designed for use by amputees in resource-poor countries. As hypothesised, the ADS was found to be significantly faster than using crutches. This is in agreement with a previous study assessing a healthy, adult population [3]. This result was maintained when using the scooter on uneven ground, which suggests that the scooter could be used effectively in the environment for which it was designed. This has implications for participation in daily life activities. In much of the developing world, there are no facilities or services for manufacture of customised prosthetic limbs. Where this is the case, amputees are forced to mobilise using crutches or sticks, making them significantly slower than their peers and inhibiting participation in daily life. This study suggests that use of the scooter could enable children to keep up with their peers, which could in turn, significantly improve their quality of life. While it was expected that the ADS would be of comparable speed to using a customised prosthetic limb, the results here indicate that the scooter was actually faster than walking with a prosthesis, and could therefore, in certain circumstances, be used in addition to, or in place of a prosthetic limb.

The ADS was also found to significantly improve energy efficiency compared to use of crutches on level ground. Improved energy efficiency implies the ability to travel longer distances, and therefore also represents potential to increase participation in individuals with an amputation. There was no difference over rough terrain, however, compared to crutch walking. This is likely due to the large inter-subject variation and small sample size. A larger cohort could potentially demonstrate improved efficiency as there was a trend towards greater efficiency while using the scooter, even on uneven ground.

The results confirm the presence of a learning effect with continued use of the ADS. It was therefore decided to use the final testing sessions (days 12 to 14) using the scooter as a comparison with crutch walking and prosthesis walking. All subjects were already proficient with use of crutches and with their prosthetic limb, so no familiarisation period was required for these conditions.

Most participants reported to find the ADS comfortable. Based on feedback from the participants, the ADS may be further improved by minor design modifications to tailor the seat and handle bars to the user. The presence of the learning curve, giving an increased feeling of stability and safety, highlights that provision of this aid, as with any mobility aid for amputees, should be part of a programme of rehabilitation and supervision to ensure that the user is fully supported when they begin using it, preferably with a trained rehabilitation expert.

Although the ADS is not currently available on the market, its conception as a functional alternative to a prosthesis, where these are not available, should be considered to promote independent mobility in lower limb amputees. Since the scooter demonstrated superior speed and energy efficiency over crutches, it appears to be a promising mobility aid. The maintenance of increased speed, even on rough terrain, suggests it is appropriate for use in developing countries.

## Future developments

The scooter was designed to offer functional mobility over a range of terrains and conditions [3]. The next phase of testing includes trials of its suitability in usual journeys including to and from school and other activities of daily living in the home environment by amputee children. This will be an important study to further clarify what structural adaptations may be required to maximise its suitability over different terrains. Potential modifications may include a higher cross bar for improved uneven ground clearance [3] or an improved collapsible mechanism to aid carrying the scooter if required due to thick, muddy terrain. The initial scooter prototypes have been costly, but the inventor is optimistic once the design has been finalised, the scooter will be mass produced for well under £50.
